# Ionic liquid-templated preparation of mesoporous silica embedded with nanocrystalline sulfated zirconia

**DOI:** 10.1186/1556-276X-6-192

**Published:** 2011-03-02

**Authors:** Antony J Ward, Ajit A Pujari, Lorenzo Costanzo, Anthony F Masters, Thomas Maschmeyer

**Affiliations:** 1 Laboratory for Advanced Catalysis for Sustainability, School of Chemistry, The University of Sydney, Bldg. F11, Sydney, NSW 2006, Australia

## Abstract

A series of mesoporous silicas impregnated with nanocrystalline sulphated zirconia was prepared by a sol-gel process using an ionic liquid-templated route. The physicochemical properties of the mesoporous sulphated zirconia materials were studied using characterisation techniques such as inductively coupled optical emission spectroscopy, X-ray diffraction, transmission electron microscopy, energy-dispersive X-ray microanalysis, elemental analysis and X-ray photoelectron spectroscopy. Analysis of the new silicas indicates isomorphous substitution of silicon with zirconium and reveals the presence of extremely small (< 10 nm) polydispersed zirconia nanoparticles in the materials with zirconium loadings from 27.77 to 41.4 wt.%.

## Introduction

Solid acids and superacids have attracted great interest due to their potential applications in many areas of the chemical industry, ranging from petrochemicals to fine chemical production [1-5]. One such example of a strong solid acid is sulphated zirconia, probably first reported in the patent literature [6] then further exemplified [7] almost two decades later. This material has shown remarkable catalytic activity for a range of reactions which are not catalysed by conventional solid catalysts [1,8,9]. Examples of such reactions include the skeletal isomerisation of alkanes [10], the alkylation of alkanes by alkenes [11], and the low-temperature cracking of polyolefins [12] and, in the presence of metal dopants, the activation (Pt) of methane [13] and (either alone or promoted by Fe or Mn) ethane [14], to name a few.

Numerous methods exist for the preparation of sulphated zirconia materials. Conventional synthetic methods include the impregnation of zirconium hydroxide with ammonium sulphate or sulphuric acid followed by calcination in air [10,11,14,15], the calcination of zirconium sulfates [16,17] and sol-gel co-precipitation [15]. Sulphated zirconias prepared by such methods and calcined at 500°C to 650°C have mesoporous structures with surface areas in the range of 70 to 190 m^2^/g. The synthesis of mesoporous zirconia using long-chained ammonium halides as the template affords a mesoporous phase of structured zirconium hydroxides with surface areas ranging between 520 and 670 m^2^/g; however, these structures are unstable above 500°C, with the mesoporous framework collapsing in the absence of the template [18-20]. Addition of dilute sulphuric acid to the material has been shown to stabilise the mesoporous structure up to temperatures of 700°C and delay the onset of crystallisation [21]. Knowles and Hudson [22] have reported the preparation of mesoporous zirconia using alkyltrimethylammonium halides (alkyl = C*_n_*, *n *= 8, 10, 12, 14, 16, 18) and, after careful drying and calcination at 450°C, obtained materials with surface areas in the range of 238 to 329 m^2^/g and *d*-spacings of the calcined materials directly depending on the length of the alkyl chain of the structure-directing agent. The preparation of sulphated mesoporous zirconia (ZrO*_x_*(SO_4_)_2 -*x*_) using alkyltrimethylammonium halides (alkyl = C*_n_*, *n *= 16, 18, 20) as the structure-directing agents and starting with Zr(SO_4_)_4_·4H_2_O resulted in materials with high surface areas (up to 390 m^2^/g de-pending on the alkyl chain length of the ammonium species). The pore structures of these materials collapsed on calcination at 500°C, but were stable on calcination at 500°C after treatment with phosphoric acid [23]. Stabilisation of the templated zirconium hydroxide with silica has been used to produce materials with surface areas of 580 to 680 m^2^/g for loadings ranging from 0.8 to 1.7 mol% Zr [24]. Pacheco and co-workers have used the anionic surfactants dodecyl sulphate and phosphate as structure-directing agents to prepare mesoporous zirconia, which, after drying at 140°C, had surface areas >400 m^2^/g [25,26]. However, the structures collapsed after calcination at 500°C.

In order to produce catalytically active sulphated zirconia possessing high surface areas, the preparation of mesoporous silica impregnated with zirconia has been undertaken by a number of research groups. The isomorphic substitution of zirconium into the mesoporous silicas MCM-41 [27-30], MCM-48 [31] and SBA-15 [32-34] has been achieved through hydrothermal Si/Zr co-precipitation in the presence of the requisite templates. El Haskouri and co-workers prepared high zirconium content MCM-41 silicas using the structure-directing agent cetyltrimethylammonium bromide [CTAB] in basic conditions to afford materials with surface areas of 907 m^2^/g (Si/Zr = 32), 997 m^2^/g (Si/Zr = 10.0), 688 m^2^/g (Si/Zr = 5.0) and 382 m^2^/g (Si/Zr = 3.0). Ying et al. have prepared MCM-41 mesoporous silica-zirconia materials using CTAB as the organic template under highly acidic conditions (pH < 1) to obtain materials with zirconium loadings up to 19.6 wt.% (S.A. = 436 m^2^/g) [35]. These mesoporous silicas impregnated with zirconia have relatively low catalytic activities which have been attributed to their relatively weak acidity [27]. A combination of [Zr(OPr*^n^*)_4_], acetic acid to suppress rapid [Zr(OPr*^n^*)_4_] hydrolysis, and CTAB as a pore-directing agent, has also been used in the synthesis of nanocrystals of tetragonal ZrO_2 _with a mesoporous structure, stable after the surfactant was removed by calcinations at 500°C [36]. Monodisperse tetragonal ZrO_2 _nanocrystals can also be prepared in a non-hydrolytic reaction between [Zr(OPr*^n^*)_4_] and ZrCl_4 _in the presence of trioctylphosphine oxide at 340°C. The nanoparticle surface is suggested to be capped with the phosphine oxide [37].

Mesoporous materials are often effective catalysts or catalyst supports. However, few catalytic reactions require that the pores be regularly ordered. Since, in the case of ZrO_2_-derived materials, regular pore ordering of mesoporous materials frequently leads to thermally unstable materials, and given the high costs of the templates used in these syntheses, a more general strategy in catalyst syntheses is to concentrate on the mesoporosity, rather than on the pore ordering, of the catalytic materials. Ionic liquids have received much interest as templating agents during the last few years [38-40], and we have recently demonstrated that ionic liquids could be used to prepare high surface area silicas with a sponge-like, three-dimensional topology [38] as well as to stabilise metal chalcoginide nanoparticles [41]. Accordingly, we reasoned that ionic liquids might be suitable media for the single-pot preparation of thermally stable, high surface area mesoporous silica-supported sulfated zirconia either as supported nanocrystals or with silicon isomorphically substituted by zirconium. We were also encouraged by the report of the incorporation of Zr into TUD-1 [42-44], although these materials had not been sulphated. It is also of interest to investigate a mesoporous metarial not prepared via the intermediacy of atranes [45]. Our investigations, and the characterisation of the new materials, are described herein.

## Materials and methods

### Experimental

#### General

##### Materials

Zirconyl chloride octahydrate, tetraethoxysilane [TEOS], tetraethylammonium hydroxide, hydrofluoric acid (48 wt.% in water, all Sigma Aldrich), boric acid, hydrochloric acid (35%, all Merck) and nitric acid (70%, Univar) were all used without further purification. Silicon (1,000 ppm) and zirconium (1,000 ppm) standards for inductively coupled plasma [ICP] analysis were obtained from Choice Analytical. 1-Hexadecyl-3-methylimdazolium bromide was prepared using a reported procedure [46].

##### Instrumentation

The nitrogen adsorption and desorption isotherms at 77 K were measured using a Micromeritics ASAP2020 Surface Area and Porosity Analyzer (Micromeritics, Norcross, GA, U.S.A.). The data were obtained by liquid nitrogen adsorption and desorption at various nitrogen partial pressures and were analysed by the Barrett-Joyner-Halenda [BJH] and the Brunauer-Emmett-Teller [BET] methods. The pore size distribution curve was derived from the analysis of the adsorption branch of the isotherm.

Powder X-ray diffraction patterns were collected on a PANalytical X'Pert PRO X-ray diffractometer (PANalytical, Almelo, The Netherlands) using Cu Kα radiation and a PIXcel solid-state detector. Low-angle diffraction experiments were conducted in a continuous mode over the range of 1° to 10° 2*θ *using a step size of 0.040° 2*θ *with a count time/step of 26 s. Wide-angle diffraction patterns were recorded in the continuous scanning mode over the range 10° to 70° 2*θ *using a step size of 0.0131° 2*θ *with a count time/step of 300 s.

Metal and silicon concentrations were determined by ICP analyses, which were performed on a Varian Vista AX ICP-AES (Varian Inc., Melbourne, Vic., Australia). The samples were prepared in the following manner. Concentrated HNO_3 _(1 mL) and HF (2.5 mL, 48 wt.%) were added to a silica sample in a polyethylene bottle (20 to 30 mg, dried for 12 h at 100°C), and this mixture was stirred at room temperature for 8 h. Then, a saturated boric acid solution (20 mL) was added. The resulting solution was then diluted to 50 mL with deionised water.

Elemental analyses were performed at the Microanalytical Laboratories at the Australian National University using a Dionex Ion Chromotography Analyzer (Dionex, Sunnyvale, CA, U.S.A).

Transmission electron micrographs were recorded digitally with a Gtan slow-scan charge-coupled device using a Philips CM120 Biotwin electron microscope (Philips, Amsterdam, The Netherlands) operating at 120 kV. The samples were prepared by dispersing the powder products as an ethanol slurry, which was then deposited and dried on a holey carbon film on a Cu grid.

High-resolution transmission electron microscopy [HRTEM] was performed using a JEOL 3000F microscope (JOEL, Tokyo, Japan) operating at 300 kV. Specimens for HRTEM studies were dispersed in ethanol, accumulated on a copper holey carbon grid and dried at room temperature. The specimens were additionally evaporated with carbon in order to prevent excessive charging and decomposition of the sample under the electron beam. During the microscope session, the objective lens voltage was adjusted to sufficiently high underfocus values (~1,000 nm).

X-ray photoelectron spectroscopy [XPS] measurements were made using a SPECS spectrometer (SPECS Surface Nano Analysis GmbH., Berlin, Germany) equipped with Al X-ray source with monochromator operating at 200 W, a hemispherical analyser and a line delay detector with nine channels. Survey spectra were acquired for binding energies in the range 50 to 1,200 eV using a pass energy of 30 eV. O*1s*, Zr*3p*_3/2 _and Si*2 s *region spectra were acquired at a pass energy of 23 eV with ten scans to obtain a higher spectral resolution (0.1-eV step) and to lower the noise level. Analysis of spectra was done using CASA software.

Catalytic reactions were monitored with a Shimadzu GC-17A gas chromatograph [GC] (Shimadzu Corp., Kyoto, Japan) fitted with an SGE capillary column (BPX5, 30 m × 0.25 mm × 0.25 μm) after calibration with mesitylene as internal standard.

##### Syntheses

Zirconium oxide was prepared by the addition of water to an ethanolic solution of zirconium butoxide such that Zr/H_2_O was 1:2. Immediately, a white gel was formed. The sample was dried and calcined at 450°C for 2 h. The surface area of this material was found to be 90.7 m^2^/g.

A typical synthesis of the mesoporous sulphated zirconia materials with various Si/Zr molar ratios was performed as follows: 1-Hexadecyl-3-methylimidazolium bromide (0.8 g, 2.23 mmol) was dissolved in aqueous dilute HCl (25 mL, 2 M, 0.05 mol) and to this solution was added TEOS (1.7 g, 8.16 mmol). The mixture was stirred at 40°C for 4 h and then the required amount of ZrOCl_2_·8H_2_O added to the stirred mixture. The resulting mixture was then stirred at 40°C for 20 h. The mixture was then transferred into an autoclave and heated at 100°C for 2 days. The pH of the synthesis gel was adjusted to 7.5 by dropwise addition of aqueous ammonia at room temperature. The resulting mixture was hydrothermally treated at 100°C for another 2 days. The final solid was collected by filtration, washed with water until no chloride was detected in the washings and dried at room temperature. The solids obtained were impregnated with aqueous H_2_SO_4 _(0.5 M, 25 mL) at room temperature for 30 min. The sulfated samples were then filtered, dried at 100°C and calcined at 550°C in air for 10 h.

### General procedure for the Meerwin-Ponndorf-Verley reductions

All reductions were performed under a nitrogen atmosphere at 80°C. SZ-SiO_2 _(**8**, 25 mg) was added to a two-necked flask prior to the addition of 2-propanol (4 mL) and mesitylene (0.1 mL, internal standard) and the reaction mixture heated to 80°C. When the desired temperature was reached, the ketone or aldehyde (2 mmol) was added. The reaction was monitored by taking small samples (~0.1 mL). The samples were centrifuged to remove the catalyst and diluted with hexane prior to analysis by GC.

## Results and discussion

**Figure 1:**
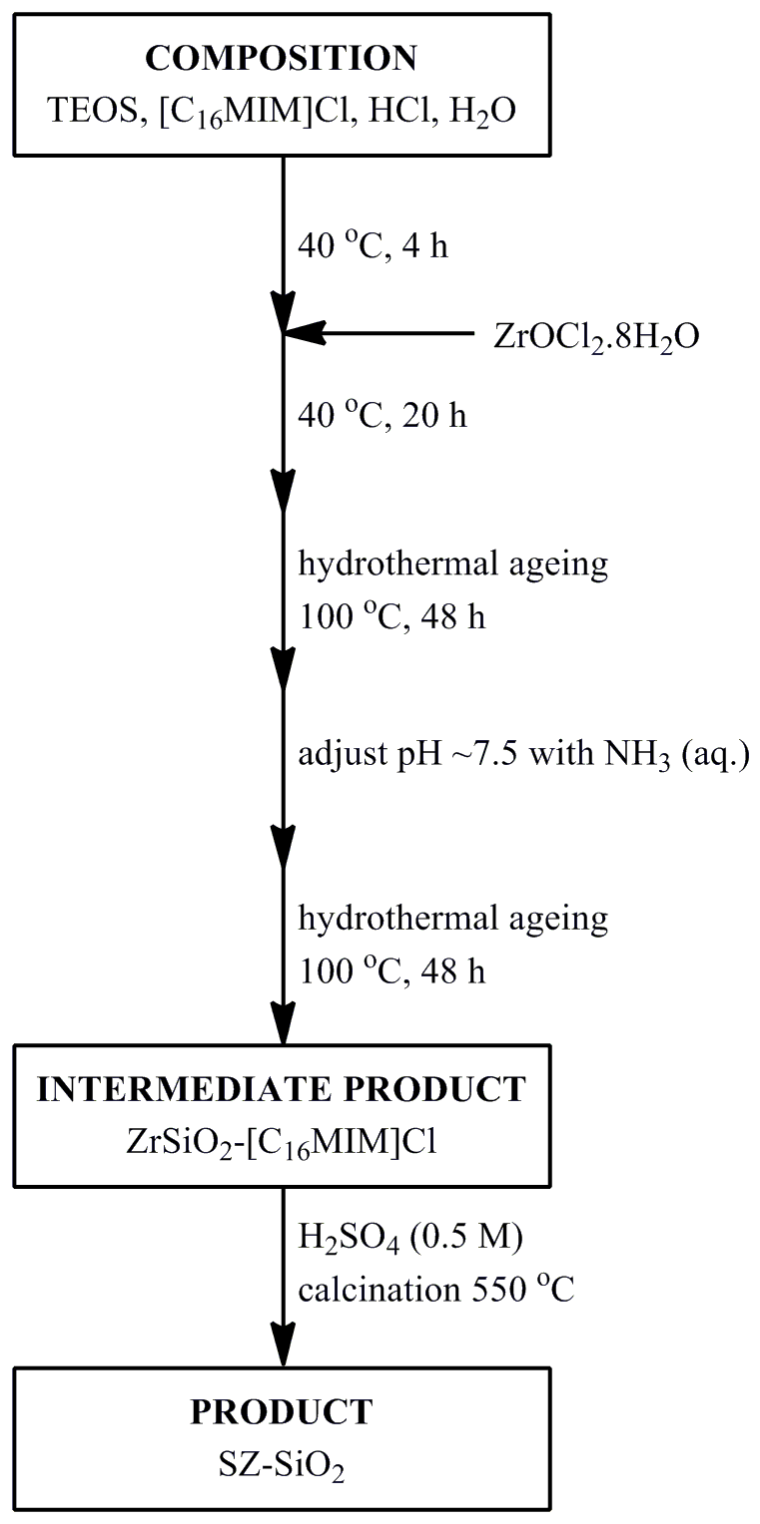
**Schematic representation of the syntheses of SZ-SiO_2 _materials**.

The mesoporous silicas impregnated with sulphated zirconia (**2 **to **9**) were prepared using a modification of the method described by Du et al. [47]. The method (schematically represented in Figure 1) involved the hydrolysis of the silica precursor (TEOS) in dilute HCl in the presence of the template 1-hexadecyl-3-methylimdazolium bromide at 40°C for 4 h, after which time the required amount of ZrOCl_2_·8H_2_O was added to achieve the desired Si/Zr ratio. In addition, a purely siliceous material (**1**) was prepared using an identical process. This synthesis differs from that of Zr-TUD-1 in that it does not proceed through silatrane intermediates [45].

Zirconium oxychloride, used in these syntheses, is present as the tetranuclear species [Zr_4_(OH)_8_(H_2_O)_16_]^8+ ^in the crystal [48] and in aqueous solution [49] at low pH. It has been shown that the more acidic the solution, the less polymerisation to higher molecular weight species occurs [50]. Hence, at pH ~1 used herein, it can be expected that the zirconium species is predominately the tetramer. From the mechanistic point of view, it can be speculated that the synthesis of mesoporous sulphated zirconia using [C_16_MIM]Br as the templating agent is mediated by the presence of Br^-^/Cl^- ^ions in the synthesis mixture, as was observed by Wong et al. [35] in the analogous CTAB templated system. In such a case, the synthesis was shown to proceed by a S^+^X^-^I^+ ^pathway [35], where S^+ ^stands for the [C_16_MIM] cation, X^- ^for the Br^- ^anion and I^+ ^for the cationic zirconium species [51,52].

Nitrogen adsorption/desorption isotherms recorded at 77 K reveal classic type IV curves, which indicate the materials to be mesoporous in nature. Such materials have pore sizes in the range of 2 to 50 nm, and type IV isotherms which exhibit hysteresis indicate that capillary condensation occurs during the adsorption process. The surface area, pore volume and pore size data of mesoporous sulphated silica-zirconia samples are presented in Table 1. The BET surface area and pore volumes show the expected decreases with increasing zirconia content: the surface areas decrease from 482 to 343 m^2^/g and the pore volumes de-crease from 1.343 to 0.4290 cm^3^/g. The silica prepared without any zirconium added had a surface area of 709 m^2^/g and a pore volume of 0.819 cm^3^/g.

**Table 1:** Physical properties of the prepared mesoporous sulphated silica-zirconia materials

Sample	Added Zr (wt.%)	**Zr loading**^a ^**(wt.%)**	Si/Zr (mol/mol)	**Sulphur loading**^b ^**(wt.%)**	Zr/S	**Surface area (m**^**2**^**/g)**	**Pore volume (cm**^**3**^**/g)**	Pore diameter (Å)
**1**	-	-	∞	-	-	709	0.819	46.15
**2**	2.2	1.01	148	0.05	7.1	435	1.343	123.52
**3**	3.6	1.34	111	0.10	4.7	416	1.291	124.23
**4**	7.9	2.43	60	0.33	2.6	482	1.166	96.89
**5**	10.9	5.29	27	0.28	6.6	470	1.075	91.47
**6**	25.0	15.35	7.8	0.73	7.4	424	1.167	110.08
**7**	39.5	26.89	3.6	1.08	8.8	378	0.763	80.73
**8**	45.0	27.77	3.4	1.22	8.0	380	0.558	58.63
**9**	61.5	41.40	1.6	1.69	8.6	343	0.430	50.23

^a^Determined by ICP-OES analysis; ^b^determined by elemental analysis.

The ratio of zirconia to silica was measured using ICP optical emission spectroscopy [ICP-OES] analysis. As seen in Table 1, there is a significant loss of the added zirconium as a result of the washing process. In the case of the SZ-SiO_2 _materials with the lowest Zr loadings (i.e. **2 **to **5**), only 31% to 49% of the added zirconium was incorporated into the final product. At the higher loadings, however, there is a significant increase in the incorporation of the zirconium: Silicas **6 **to **9 **have incorporation levels between 61% and 69%. As a consequence, zirconium loadings range from 1.01 to 41.40 wt.%.

Sulfur loadings were determined by elemental analysis of the prepared materials. As expected, the sulfur content of the materials increases with zirconium loading (Table 1). In the case of **5**, the sulfur loading is lower than expected and may indicate the presence of larger ZrO_2 _domains, compared to that of **4**, which has resulted in a lower available surface area for sulfate coordination. However, the amounts of sulfur are low for all materials, indicating that the concentration of zirconium at the surface is low. In this work, the highest sulfur loading is 1.69 wt.% for material **9 **which has a zirconium loading of 61.5 wt.%. The sulfur loadings for the sulfated zirconia-silica materials prepared by Du and co-workers range from 1.96 to 3.88 wt.% for zirconium contents ranging from 20.1 to 33.8 wt.% [34].

**Figure 2:**
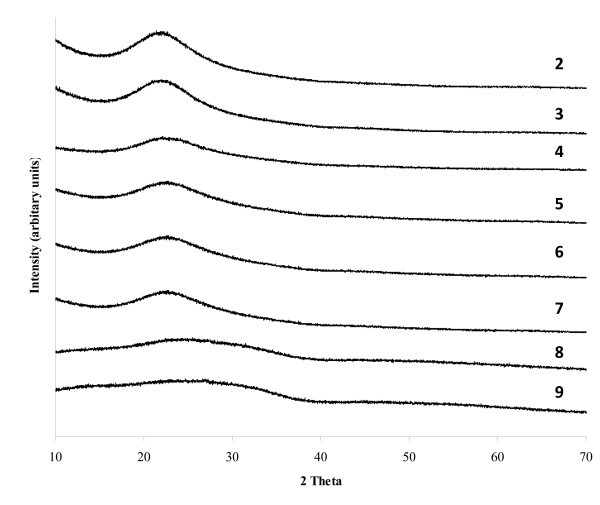
**High-angle X-ray diffraction patterns of the prepared sulphated zirconia-silica materials**.

The lack of a significant peak at low angle suggests little long-range mesostructure in the material [21,53]. The diminution in peak intensity compared to the purely siliceous material **1 **is attributable to the decrease in pore size and the filling of the mesopores with zirconium oxide nanoparticles at the highest loadings. The absence of additional absorptions at low angle indicates that there is no ordering within the silica. The high-angle (2*θ *> 10°) X-ray diffraction patterns of the mesoporous SZ-SiO_2 _materials **2 **to **9 **(Figure 2) reveal a broad peak from 20° to 35° 2*θ *which is due to the amorphous nature of the silica support. In the case of **8 **and **9**, this peak is significantly broadened compared to that observed for materials **2 **to **6**, and this broadness may be due to the significant amounts of zirconium that have been incorporated into the silica framework. In all cases, there is no diffraction associated with a crystalline phase of ZrO_2_, indicating no bulk crystalline zirconia is present.

XPS was used to probe the surface composition of the SZ-SiO_2 _materials. As seen in Table 2, the Zr surface concentrations for materials **2 **to **7 **are relatively low, indicating that the bulk of the zirconia is located within the silica matrix. The relative concentrations of zirconia increase considerably for silicas **8 **and **9 **which possess ZrO_2 _nanocrystallites on the surface of the silica (*vide infra*). Assuming that the silicon present within the samples is present as silica and setting the Si*2p *binding energy at 103.6 eV (the value for SiO_2_) [54], then the binding energy for Zr*3d*_5/2 _has a corrected value between 182.8 and 183.7 eV. These values are higher than for bulk ZrO_2 _(182.2 eV) and closer to that of ZrSiO_4 _and of Zr in Zr-TUD-1 (183.3 and 183.2 eV, respec-tively) [44,55]. Similarly, the O*1 s *values are in the range of 531.7 to 532.3 eV, which are significantly higher than the binding energy seen in ZrO_2 _but approximate those in Zr-TUD-1 and in SiO_2 _(530.2, 532.4 and 532.9 eV, respectively) [44,55]. The use of XPS to probe the S*2p *binding energy was not possible due to the low sulfur loadings (as determined by elemental analysis), which resulted in the observed peak having poor signal-to-noise ratio.

**Table 2:** Relative concentrations of O, Zr and Si (%) at the surface of the SZ-SiO_2 _materials as determined by XPS

	2	3	4	5	6	7	8	9
O*1s*	68.26	69.63	75.91	72.08	78.33	69.49	84.03	74.58
Zr*3p*_3/2_	0.22	0.24	0.21	1.20	1.02	1.28	5.69	9.00
Si*2s*	31.53	30.14	31.53	26.72	20.65	29.23	10.28	16.43

**Figure 3:**
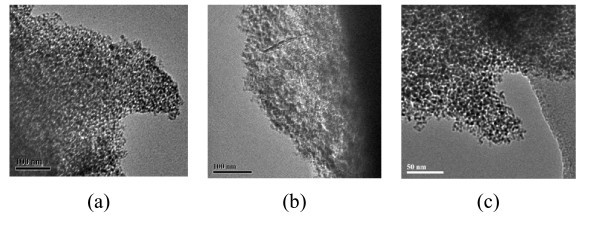
**Representative TEM images of the prepared SZ-SiO_2 _samples**. **a **Silica **3 **(1.34 wt.% Zr). **b **Silica **4 **(2.43 wt.% Zr). **c **Silica **8 **(27.77 wt.% Zr).

The TEM images of the SZ-SiO_2 _samples **3**, **4 **and **8 **are shown in Figure 3. As seen from these images, silica does not possess long-range order, which is consistent with our observations when using similar templates to produce high surface area mesoporous silica [38]. In addition, no large clusters of nanoparticulate zirconia are visible at the magnifications used, which indicates that the zirconia is present as either very small nanoparticles or incorporated into the silica framework.

**Figure 4:**
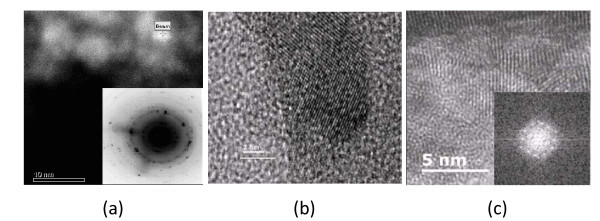
**HRTEM images of the prepared SZ-SiO_2 _samples 7 to 9**. **a **Dark field image of silica **7 **(26.89 wt.% Zr). **b **Silica **8 **(27.77 wt.% Zr). **c **Silica **9 **(41.40 wt.% Zr).

The dark field and HRTEM images of several of the sulphated zirconia-silica samples are shown in Figure 4. Dark field images show strong atomic number (*Z*) contrast, thus allowing heavier elements to be visualized. Hence, the dark field image of **7 **(Figure 4a) shows the dispersion of the Zr atoms within the silicon matrix and is representative of similar images obtained for materials **8 **and **9 **(not shown). In all of the TEM images, small crystalline particles associated with the walls of the silica surface are observable. In the case of silica **7**, it was very difficult to observe the very small crystalline ZrO_2 _particles in the HRTEM, but careful analysis of the electron diffraction pattern clearly indicates that polydispersed ZrO_2 _particles are present. From the diffraction pattern, it is evident that the material is polycrystalline with some crystal orientation evident. The HRTEM images of **8 **and **9 **clearly show the crystalline phase of ZrO_2 _on the surface of the silica, with the particles being in the size range of 5 to 10 nm.

Preliminary screening of the catalytic activity of these materials was probed using the Meerwin-Ponndorf-Verley reductions [56] of aldehydes and ketones as a test reaction and SZ-SiO_2 _(27.77 wt.% Zr (**8**)) as a representative material, containing both nanoparticulate SZ-SiO_2 _and framework zirconium. This reaction and its reverse, the Oppenheimer oxidation, have been catalysed by calcined hydrous zirconium oxide [57-60]. The results using SZ-SiO_2 _(**8**) are collected in Table S1 in Additional file 1.

Sample SZ-SiO_2 _(**8**) catalyses the Meerwin-Ponndorf-Verley reductions of a representative aldehyde (benzaldehyde) and a ketone (acetophenone). The conversions are comparable to those using Zr-TUD-1 as catalyst [42]. The activity of the catalyst appears to correlate with the p*K*_HB _of the substrate [61], inasmuch as substrates with p*K*_HB _> 1.10 are inactive. Although we observe no detectable reaction of cinnamaldehyde with 2-propanol over 20 h, this reaction is slow, even with ZrO_2 _catalysts of 'moderate activity' [62].

## Conclusions

This study has shown that 1-hexadecyl-3-methylimidazolium bromide can be successfully used as the structure-directing agent in the synthesis of mesoporous sulphated zirconia. The materials, prepared with varying ratios of zirconium to silica, displayed surface areas in the range of 343 to 482 m^2^/g and mesopore volume ranging from 0.43 to 1.34 cm^3^/g. This method of preparation of the SZ-SiO_2 _materials for loadings up to 41 wt.% Zr resulted in the formation of nanocrystalline zirconia in the silica host, with the zirconium also present as polydispersed nanoparticles of ZrO_2 _on the surface at the higher loadings. The materials are active in the catalysis of the Meerwin-Ponndorf-Verley reductions of aldehydes and ketones with p*K*_HB _values <1.1.

## Competing interests

The authors declare that they have no competing interests.

## Authors' contributions

AJW prepared and characterized materials and performed catalytic studies. AAP prepared and characterized materials. LC recorded TEM images. AFM and TM conceived of the study, and participated in its design and coordination. All authors read and approved the final manuscript.

## Supplementary Material

Additional file 1 **Table S1**. Meerwin-Ponndorf-Verley reductions catalysed by SZ-SiO_2 _(27.77 wt.% Zr (**8**))^a^. Click here for file
