# Supplementary material for: Ionic liquid-templated preparation of mesoporous silica embedded with nanocrystalline sulfated zirconia
Source: Nanoscale Res Lett. 2011 Mar 2;6(1):192. doi: 10.1186/1556-276X-6-192 (PMC3211248; doi:10.1186/1556-276X-6-192)
Supplement: Additional file 1 — Table S1. Meerwin-Ponndorf-Verley reductions catalysed by SZ-SiO2 (27.77 wt.% Zr (8))a. [file 1556-276X-6-192-S1.DOC]

Table S1. Meerwin-Ponndorf-Verley reductions catalysed by SZ-SiO_2_ (27.77 wt.% Zr (**8**)).*^a^*

| **Substrate** | **Products** | **p*K*_HB_*^b^*** | **Conversion (%)***^c^* | **Reaction time (h)** |
| --- | --- | --- | --- | --- |
|  |  | 0.78 | 48  (37% (**A**); 21% (**B**))*^d^* | 96 |
|  |  | 1.10 | 9 | 96 |
|  |  | 1.13 | 0 | 20 |
|  |  | N/A | 6 | 96 |
|  |  | N/A | 0*^e^* | 96 |

*^a^* Reaction conditions: 25 mg catalyst, 4 mL 2-propanol, 2 mmol substrate, 100 μL mesitylene (internal standard), 80 ^o^C, performed under N_2_ atmosphere. *^b^* Values of p*K*_HB_ obtained from reference.^61^ *^c^* The products were identified using GC-MS. *^d^* The formation of **B** results from the reaction of the generated alcohol and subsequent reaction with one equivalent of the starting aldehyde to form a hemiacetal and the resulting hemiacetal reacting with the solvent to form the observed acetal. *^e^* No products from the reduction of the aldehyde were seen, however, products resulting from the reaction of the aldehyde with the solvent (*ie*. hemiacetal and acetal) were observed.
